# Relationship between Dietary Polyphenols and Gut Microbiota: New Clues to Improve Cognitive Disorders, Mood Disorders and Circadian Rhythms

**DOI:** 10.3390/foods12061309

**Published:** 2023-03-19

**Authors:** Siyu Liu, Lu Cheng, Yanan Liu, Shengnan Zhan, Zufang Wu, Xin Zhang

**Affiliations:** 1 Department of Food Science and Engineering, Ningbo University, Ningbo 315211, China; 2 Department of Food Science, Rutgers, The State University of New Jersey, New Brunswick, NJ 08901, USA

**Keywords:** intestinal flora, neurodegenerative diseases, dietary polyphenols, gut–brain axis, interaction

## Abstract

Cognitive, mood and sleep disorders are common and intractable disorders of the central nervous system, causing great inconvenience to the lives of those affected. The gut–brain axis plays a vital role in studying neurological disorders such as neurodegenerative diseases by acting as a channel for a bidirectional information exchange between the gut microbiota and the nervous system. Dietary polyphenols have received widespread attention because of their excellent biological activity and their wide range of sources, structural diversity and low toxicity. Dietary intervention through the increased intake of dietary polyphenols is an emerging strategy for improving circadian rhythms and treating metabolic disorders. Dietary polyphenols have been shown to play an essential role in regulating intestinal flora, mainly by maintaining the balance of the intestinal flora and enhancing host immunity, thereby suppressing neurodegenerative pathologies. This paper reviewed the bidirectional interactions between the gut microbiota and the brain and their effects on the central nervous system, focusing on dietary polyphenols that regulate circadian rhythms and maintain the health of the central nervous system through the gut–brain axis.

## 1. Introduction

The relationship between gut microbes and the brain has become one of the most exciting but controversial topics in microbiome research. The gut microbiota is integral to neurodevelopmental, physiology, psychology and ethology-related processes. The gut microbiota is closely linked to the host’s central nervous system through the nervous, endocrine, immune and metabolic systems [1]. The information communication channel involved in the interaction between gut microbiota and the neuronal system is called the gut–brain axis (GBA). A series of information transmissions between various systems can directly or indirectly affect cognition, emotion, sleep, learning ability, and other brain functions [2].

The gut microbiota itself is considered an important bridge between diet and human health. Intestinal microbiota diversity provides biochemical functions not possessed by the body’s enzymes, such as promoting intestinal activity, affecting nutrient absorption, participating in host conversion, and protecting the intestinal epithelial barrier against external infection. Disturbances in the composition and levels of intestinal flora can cause the accumulation of harmful toxins in the body and affect the secretion of neurotransmitters, thus disrupting the intestinal barrier and the blood–brain barrier (BBB), causing irritable bowel syndrome, depression, anxiety, multiple sclerosis, Alzheimer’s disease (AD), diabetes, autism, etc. [3]. The GBA is a bidirectional information regulatory pathway composed of the central nervous system (CNS), neuroendocrine system, neuroimmune system, autonomic nervous system and enteric nervous system. The intestinal microbiota mainly affects CNS diseases by affecting the inflammatory response, metabolites of intestinal microbiota, the hypothalamic–pituitary–adrenal (HPA) axis, and the vagus nerve (VN) [4]. Differing from traditional treatment, changing the composition and abundance of gut microbiota has become a new way of intervening in CNS abnormalities.

Phenolic compounds are the general term for plant components with several phenolic hydroxyl groups in their molecular structure. Polyphenols are classified by structure into two major groups: flavonoid and non-flavonoid compounds. The former includes anthocyanins, flavonols and flavanones, among others; the latter includes small-molecule phenolic acids such as caffeic, ferulic and chlorogenic acids and positive trans-resveratrol, among others. Phenolic compounds from food are called dietary polyphenols. They are important functional components of plant foods that are widely found in fruits, vegetables, cereals, tea, coffee and other foods [5]. Numerous studies have shown that dietary polyphenols have a preventive and controlling effect on human ageing and metabolic diseases. In recent years, further study of dietary polyphenol morphology and gut microbes has led to new insights into the metabolic absorption of polyphenols. Dietary polyphenols can be divided into free polyphenols and binding polyphenols, according to their morphology. Free-state polyphenols are mainly absorbed in the small intestine, while binding-state polyphenols need to be used in the human metabolism under the action of a series of enzymes produced by gut microorganisms [6]. At the same time, dietary polyphenols and their metabolites were found to have a significant effect on the bacterial phase of gut microorganisms [7]. Dietary polyphenols, such as curcumin, tea polyphenol and resveratrol, have good antioxidant and anti-inflammatory effects and are often used to prevent age-related cognitive decline and neurodegenerative diseases [8].

There has recently been increasing interest in identifying bacteria that convert polyphenols in the gut. Several groups of bacteria that catalyse the metabolism of phenolics and their catabolic pathways have been demonstrated [9]. Most genera involved in converting polyphenols are *Bifidobacterium*, *Bacillus*, and *Firmicutes*. Microorganisms are influenced by external factors (e.g., diet, drugs and physical exercise), the host’s geographical distribution and individual differences [10]. Age-related changes in the composition of the intestinal flora may affect the bioavailability of certain nutrients, including their metabolically active mediators. Specific metabolic phenotypes produce biologically active metabolites that have health effects, may also reflect the composition and metabolic state of the gut microbiota and may serve as biomarkers of the gut-microbiota-mediated health effects of polyphenols [11].

Dietary polyphenols also act as prebiotics. The ingestion of plant polyphenols, especially catechins, anthocyanins and proanthocyanins, increases the abundance of *Lactobacillus*, *Bifidobacteria*, *Ackermannella*, *Roses* and *Faecobacteria*. In addition, supplemental dietary polyphenols promote the production of short-chain fatty acids (SCFAs), including butyrate. Dietary polyphenol intake cannot only regulate the composition of gut microorganisms and optimize function. It can also strengthen the link between the GBA and the CNS, thereby regulating cognitive and emotional brain functions and relieving or curing neurodegenerative diseases [12].

In recent years, the relationship between the gut microbiota and neuropsychiatric diseases has become a research hotspot. The exploration of some neuropsychiatric disorders has gradually shifted from targeting the CNS to targeting changes in gut microbes, gut flora and the GBA. This study reviews the bidirectional role of dietary polyphenols and intestinal flora, focusing on the GBA’s mechanisms of action in improving cognitive impairment and circadian rhythm disorders from a metabolic, anti-inflammatory and immunomodulatory perspective. This paper also provides a comprehensive overview of dietary polyphenols and the impact of their bioavailability conversion in the interaction with the intestinal flora on human health, offering new perspectives on the selection and intake of dietary polyphenols in conjunction with the intestinal microbiota.

## 2. Mechanisms of Action of Gut Microbiota Intervention in the Central Nervous System

### 2.1. Gut Microbiota Modulates Mood through Neural Pathways

Currently, an increasing number of studies are focusing on the relationship between gut flora and psychiatric disorders and their possible mechanisms. As a key regulator of the gut–brain axis, the gut flora has emerged as an important factor in the development of depression [13]. The structure of the gut flora is significantly altered in patients with depression compared to healthy controls [14]. Animal studies have shown that gut flora dysbiosis leads to an abnormal stress response in the host, reduced neurogenesis and increased neuroinflammation. The transplantation of faeces from depressed loyalists into germ-free mice induced behavioural and physiological features of depression, including a lack of pleasure and depressive-like behaviour [15]. Furthermore, altered intestinal flora may cause increased intestinal barrier permeability, the activation of systemic inflammatory and immune responses, and the modulation of the release of the monoamine neurotransmitter S-HT, altering the activity and function of the HPA axis and levels of BDNF, ultimately leading to depression [16]. Disrupting the gut flora in healthy individuals, for example, increases depression. Rebalancing the intestinal flora (e.g., faecal transplantation, healthy diet, probiotics, prebiotics, exercise or medication), on the other hand, can have an antidepressant effect [17].

An analysis targeting neural pathways that regulate emotions showed three main pathways in the GBA to regulate the interaction between the gastrointestinal system and the brain nervous system: the neurological endocrine pathway, the immune regulatory pathway and the VN pathway. The VN is a neural pathway composed of sympathetic fibre bundles which can connect the gastrointestinal system with the nervous system in the GBA. The VN is transmitted to the end of the neurotomy, involving histamines, 5-HTs, prostaglandins, and cytokines [18]. The VN pathway is not directly related to the intestinal microbial group but instead determines brain activity by transmitting sensor signals between the intestinal microbial metabolites, dopamine and amino acids [19]. Intestinal microorganisms may inhibit the growth and development of T cells in the intestinal lymph tissue in the immune system, causing intestinal inflammation and affecting neurological diseases through VN such as depression, anxiety, and autism [20]. The intestinal flora can also affect the endocrine system of the brain through the hypothalamus–pituitary–adrenal axis (HPA), stimulate the CNS and cause anxiety and other emotional disorders. Through measuring the concentration of norepinephrine (NE) and aldosterone (ALD) with different intestinal microorganisms, the conclusion is that the intestinal flora affects the endocrine system through HPA [21].

The BBB and intestinal barriers are key components in brain control and intestinal flora regulation. The exchange of molecules and nutrients between the BBB control the circulation system, and the brain substance plays a role in maintaining the stability of the CNS [22]. The intestinal barrier can protect the intestinal mucosa by secreting SCFAs and helps the growth of intestinal epithelial cells, maintaining the stability of the intestinal system. At the same time, intestinal microorganisms will affect the host’s γ-aminobutyric acid (GABA) system. The loss of brain-derived neurotic nutritional factor (BDNF) will affect the CNS, causing depression and hindering neurological development, synapse formation, neuron survival, cell differentiation and other processes, which will cause nervous system injury [23].

Many studies have found that exploring the mechanisms of action between the nervous system and the gut microbiota has focused more on the gut microbiota’s modification of the nervous system [24]. Intestinal microorganisms can affect the colour’s normal metabolic and movement levels. Syminopine is a crucial substance in the entire brain–intestine axis system, and it also prefaces the main body of 5-hydroxytryptamine (5-HT) secretion and metabolism [25]. SCFAs can stimulate the intestinal enterochromaffin cells to produce 5-HT, affecting the brain’s emotions and changing the metabolism of 5-hydroxylidin in the CNS. Therefore, the lack of 5-HT is a crucial feature of the depression pathogenesis mechanism [26].

Emotional disorders such as depression, anxiety and autism will also, in turn, affect the richness and diversity of the intestinal microbial group [27]. For patients with depression, the levels of mobilization and alcohol in the body increase, and the level of thick-wall bacteria decreases. For patients with autism, the level of thick-wall bacteria and deformation bacteria in the body increases, and the level of bacteria decreases [28]. The changes in the intestinal microbial groups of autism patients are also related to the level of neurotoxins generated. Neurotoxins increase the expression of C-FOS in VN sensory ganglia and inhibit the release of neurotransmitters [29].

### 2.2. Mechanisms of Gut-Flora-Induced Cognitive Impairment

With an increase in age and inflammatory response, a decreased antioxidant capacity, BBB damage, and hippocampus structural changes are related to the decline in cognitive function caused by ageing. Cognitive dysfunction includes dementia, amnesia and delirium. Patients cannot perceive information about time and space, and the back of the brain cannot perceive and analyse data. It is difficult for patients to establish information models. Information models can be divided into perception, memory and thinking [30]. Intestinal flora can affect cognitive functions through the GBA. The intestinal flora affects brain memory, concentration, emotion and other brain functions through the neurotransmitter, VN, neurobiological endocrine, immune regulation, and other channels. It also affects the cognitive function of β-starch-like protein deposition, fat polysaccharide levels, and the development of small glue cells. Disorders of the intestinal flora can cause damage to cognitive function. The intestinal flora and metabolites of patients with cognitive impairment, that is, abnormal metabolites, stimulate external immune inflammation, promote peripheral inflammatory immune cells into the brain-induced inflammation and seriously affect cognitive ability [31].

The nerve transmission of intestinal flora stimulation (GABA, 5-HT, glutamic acid (GLU), NE, dopamine, etc.) can directly stimulate the CNS to generate signals. Among them, metabolic by-products generated by the flora can also interact with adrenaline to stimulate the hypothalamus to generate BNDF, which further affects cognitive ability. It is worth noting that the materials (such as serum, amino acids and GLU) produced by the intestinal microorganisms can also be used as information transmission media to coordinate communication between the systems in the body [32]. Among them, GLU stimulates the CNS, and GABA exists in many immune cells which suppress the CNS by regulating the immune system [33]. The horizontal influence of GABA on the intestinal flora affects the VN system, and GABA further plays a role in preventing neurological diseases [34]. GLU and GABA are neurotransmitters that stimulate different areas of the CNS, including the cerebral cortex, base nerve section, edge system and hypothalamus [35]. Intestinal microorganisms may affect the GLU/GABA ratio in the intestinal cavity, thereby affecting the transition of the GBA’s mid-intestinal signal. During the ageing process, small glue cells are activated during the development of the CNS. Small glue cells are tissue macrophages of the CNS that are mainly responsible for repairing damaged neural networks [36]. Microglia can increase β-secretase and γ-secretase activity and contribute to β-amyloid accumulation, creating a disruptive feedback loop that leads to neuronal interactions, leading to the remodelling of dendritic spines during synaptogenesis [37]. The occurrence of inflammatory cytokines that lead to increased inflammation may be related to sepsis [38]. Inflammatory cytokines destroy the hippocampal neurons by reducing the dendritic branches of the hippocampus. The hippocampus is an integral part of the brain’s ability to learn, cognition, and memory in the CNS. In ageing groups with cognitive obstacles, the hippocampus will show signs of shrinking and significant atrophy. Therefore, most studies have speculated that the atrophy of the hippocampus is also a mechanism that induces severe cognitive impairment. The synapse structure of the hippocampus CAL region has changed, reducing the speed of neurotransmitting and the transmission and recession of the brain’s storage information capacity, thereby increasing the degree of cognitive obstacles for ageing people. Many studies have built mouse models to compare the degree of intervention of mouse mental obstacles. Compared with the control mice, AD mice in a sterile environment have less amyloid β-protein [39]. Wang et al. checked the permeability of the BBB in living and disease-free primary mice. Their results showed that the permeability of the BBB in the body of sterile mice increased, and the expression of proteins changed [40]. However, the increase in the number of normal intestinal flora in infertile mice will lead to a decrease in the BBB permeability, and the reduction in the BBB permeability causes the loss of nutrients and cannot ensure the balance and stability of the neurons. This can easily lead to the accumulation of amyloid β-protein, thereby aggravating cognitive obstacles. These factors indicate that intestinal microorganisms play a role in the starch and protein changes in mental barriers. Heijtza et al. compared the behavioural activity and neurotransmitters of mice without specific pathogens and mice without bacteria. Early exposed intestinal microorganisms reduced the expression of PSD-95 and synapses in the two types of mouse pattern. The fungal mice demonstrated increasing exercise and reduced anxiety, which is related to the long-term enhancement of the cerebellum [22]. These results show that the microbial regulation process of host physiology affects the neural circuit signal mechanism that affects anxious behaviour. Mahmoudiandehkordi et al. also found a strong interaction between AD and the intestinal system. Concentrations of secondary bile acids, deoxycholic acid and their conjugates are consistently increased in AD. As bile acid levels increase, cognitive impairment in the host becomes more severe [41].

**Figure 1:**
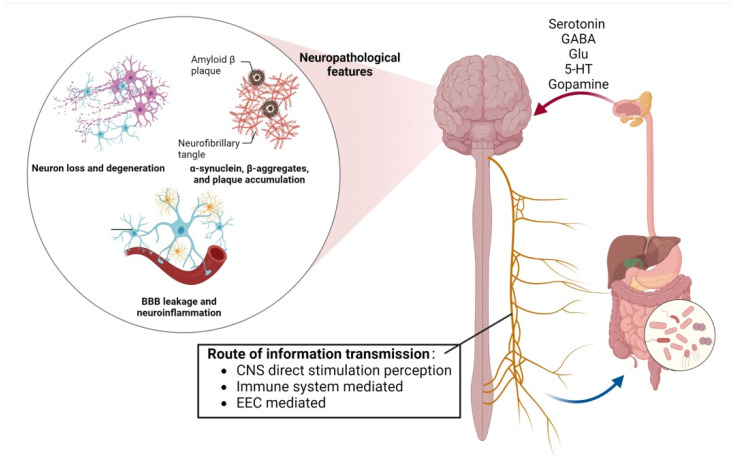
The bidirectional action between the gut–brain axis can be regulated by the blood–brain barrier, neurons and amyloid β-protein, affecting the emotional and cognitive parts of the brain.

The conclusion that sleep deprivation affects cognitive ability has also been verified in recent years. After 40 hours of sleep deprivation, healthy adult subjects have demonstrated reduced immunity, increased inflammation, and severe impairment to cognitive functions such attention, memory and other cognitive functions. To further prove that the sleep deprivation of intestinal flora affects cognitive function, between sterile mice and ordinary mice, the cognitive ability of the sterile mice was found to be less damaged. Cognitive function was significantly impaired in germ-free mice after faecal transplantation. This series of animal experiments and clinical studies suggest intestinal flora disorders may mediate sleep-deprivation-induced cognitive impairment (Figure 1) [42].

### 2.3. Intestinal Flora Interacts with Circadian Rhythms through the GBA Bidirectional Circulatory System

The GBA is a two-way communication chain between the four systems (endocrine system, intestinal system, endocrine system and CNS) [43]. The regulatory factor in the system can affect the interaction between each system and, through the GBA’s transmission, regulate the steady state of the host microbial group. Microorganisms produce metabolites in the gastrointestinal system, and SCFAs can cross the BBB to affect the growth of neurons and synapses in the hippocampus and amygdala, interacting with sympathetic nerves and nerve cells [44].

Successive sleep plays a crucial role in maintaining physical functions, and lack of sleep is a driving factor in the cause of various diseases. A host with intestinal disease has abnormal levels of reactive oxygen species (ROS), which may cause inflammation by destroying DNA and the immune system [45]. In the absence of sleep, there is a large amount of ROS accumulation in the intestine. ROS are unstable, vital oxidation short-cycle molecules [46]. The valence electron in a large amount of ROS is unpaired and unstable; therefore, it depends on oxygen and has a strong oxidation ability. It is easy to deprive other macromolecules of electrons to meet their needs. ATP is also required for the oxidation of ROS, and it is well-known that ATP increases in the waking state to meet the needs of ROS in large quantities. In contrast, sleep, as a traditional metabolic regulation process, will put pressure on the endoplasmic reticulum, inhibit the massive production of mitochondrial ATP, inhibit the accumulation of ROS and protect the CNS [47]. In fly experiments conducted by Vaccaro et al., sleep-deprived flies died rapidly. When sleep-deprived flies were fed antioxidant compounds or gut-targeted antioxidant enzymes, the survival rate of flies that prevented the accumulation of ROS was significantly higher [48]. Kempf et al. also found that sleep deprivation altered the REDOX state of sleep-regulating neurons in flies, affecting the CNS activity [49]. Conditional phosphate in the brain is caused by sleep, affecting day and night rhythm. If sleep is interrupted, phosphoric acidization will gradually decrease [50].

Biological rhythms exist in all living organisms, including circannual rhythms and circadian rhythms, and are closely related to metabolism and nutrition, affecting the activity of bioactive compounds [51]. Recently, the interaction of circadian rhythms with the gut flora has become key to the study of the metabolic regulation of the organism [52]. The day and night rhythm is a physiological shock process produced by environmental synchronization and endogenous creature clocks [53]. The day and night rhythm consists of two systems (central and peripheral clock systems) which affect the expression of most genes in the body [54]. The central clock system, located in the suprachiasmatic nucleus (SCN), is an oscillator of circadian rhythms. It passes the light to the SCN through retinal stimulation to regulate neuron and blood circulation under dark conditions [55]. The molecular rhythm clock comprises CLOCK and BMAL1 activists and PER and CRY blocking agents. The rhythm of day and night is driven by negative feedback between factors [56]. By improving the composition of the intestinal flora, the rhythm of day and night participates in various physiological reactions (including nutritional absorption, energy regulation, glucose metabolism and immune system regulation) [57]. The intestinal microorganisms themselves also affect rhythm of the host. Despite different environmental factors, the expression of clock genes in sterile mice due to a lack of intestinal microbial groups will also be damaged [58]. Most disease mechanisms are exposed when the central endogenous clock (liver clock) is destroyed. Moreover, liver clocks are not only affected by time but also by eating habits [59]. When the rhythm of day and night is destroyed, the harmful bacteria (such as inflammatory bacteria) in the intestinal microorganisms will increase. In contrast, beneficial bacteria (anti-inflammatory and SCFAs) will be reduced. Long-term rhythmic disorder affects the brain’s normal development, destroys the bodily environment’s steady state, and causes the host to suffer from depression and bipolar mood disorders. It also hurts memory and cognitive functions through the CAMP Map Mapk CREB pathway, which seriously harms health [60]. The interaction between the host’s circadian rhythm and gut flora is bidirectional. The gut flora affects the host’s energy metabolic system by regulating the efficiency of energy extraction from food and the way in which energy is obtained [61]. Oxidation-related substances, such as enzymes, ROS and oxidants, also significantly affect the stability of circadian rhythms [62]. Melatonin has solid oxidative properties that remove ROS and, due to its lipophilic nature, it is readily involved in interactions between the brain and the gut microbiota via the BBB [56]. The loss of the gut microbiota affects the activity of rhythm promoters and terminators, altering gene expression and ultimately reprogramming circadian chromatin [63]. In contrast, circadian arrhythmias also provide negative feedback to the gut flora, increasing the permeability of the gut barrier and leading to an abnormal metabolism and even endotoxemia [64].

## 3. Biphasic Action of Dietary Polyphenols and Intestinal Flora to Intervene in Neurocentral Disorders

### 3.1. Metabolic Regulation of Dietary Polyphenols by Intestinal Microorganisms

Dietary polyphenols are a type of secondary metabolite with a polyphenol structure which can be divided into phenolic acids (a hydroxybenzoic acid derivative and a hydroxycinnamic acid derivative), flavonoids (flavonols, flavanols, flavanones, isoflavones and anthocyanins), diphenylene and lignin. The bi-directional interaction between dietary polyphenols and intestinal microflora results in the biochemical transformation of the initial compounds into metabolites with higher bioavailability. The three main catabolic processes involved in the biotransformation of polyphenols by intestinal microorganisms are hydrolysis, cleavage and reduction reactions. Following these catabolic reactions, the released aglycon may undergo a phase II metabolic process and be converted by the intestinal microbiota into simple phenolic derivatives, thereby facilitating their absorption by the body [65]. Most of the polyphenols we ingest cannot be absorbed in the small intestine, and these polyphenols accumulate in the large intestine. Intestinal enzymes then metabolize them into a series of low-molecular-weight polyphenol metabolites that the gut can absorb, thereby conferring health benefits [66]. Some studies have reported the release of rhamnose from flavonoid rhamnosides by *Lactobacillus* and *Bifidobacteria*. Other intestinal bacteria, such as *Bacillus*, Enterococcus and *Enterobacter*, also show this activity. For example, rutin and diosmin are hydrolyzed by *Enterococcus*, Bacteroides, Paracoccus and *Brautella*, resulting in the release of quercetin and diosmin as their glycosides [67].

Cleavage, as the most critical catabolic step, includes opening the carbon ring, breaking the CC bond and removing the methyl ether. Gut bacteria and members of Coriobacteriaceae perform these functions. The glycosides released after flavonoid hydrolysis are catabolized by C-ring cleavage. After the catabolic reaction, the released glycosides may undergo a phase II metabolic process and be converted into simple phenolic derivatives by the gut microbiota, thereby facilitating bodily absorption [68]. The primary mechanism by which intestinal microorganisms mediate the metabolic degradation of polyphenols is due to the presence of bound polyphenols, which are mostly covalently bound to structural components of the cell wall (e.g., cellulose, hemicellulose, lignin, pectin, proteins, etc.) and are able to resist digestion by the stomach and small intestine to reach the colon [69]. The intestinal flora in the colon can secrete a variety of polyphenol metabolases (α-rhamnosidase, β-glucosidase, β-glucuronase, etc.). These enzymes catalyse the deglycosylation of glycosidic polyphenols, the dissociation of proanthocyanidin polymers and the hydrolysis of esterified phenolic acids. These reactions culminate in dissociating the bound polyphenols and releasing them as free polyphenols. After intestinal microorganisms have freed dietary-bound polyphenols, the free polyphenols that enter the colon directly can be further metabolized and degraded by the action of intestinal microorganisms [70]. The metabolic degradation of free polyphenols by intestinal microorganisms is mainly achieved through the action of a series of enzymes secreted by the intestine. Couteau et al. isolated *E. coli*, Bifidobacterium and *Lactobacillus gasseri* from faeces that produce cinnamoyl esterase, which catalyses the breakage of the phosphodiester linkage of chlorogenic acid to release caffeic acid [71]. Glycosides (e.g., quercetin) formed with rhamnose, arabinose and xylose can only be further digested and absorbed if they reach the colon and are degraded by the action of rhamnosidase (produced by intestinal microorganisms) and glycosides are released [72]. Metabolic processes may produce metabolites that contribute to intestinal metabolic homeostasis, including organic acids (e.g., lactate, pyruvate and succinate), gases (e.g., H_2_, H_2_S and CO_2_), and SCFAs (e.g., acetate, propionate and butyrate). The result is a moderate decrease in the colonic pH and a reduction in nitrogenous end products and faecal enzymes [73].

The different reduction reactions of polyphenols (e.g., double-bond hydrogenation, carbonyl reduction and dehydroxylation) are catalysed by intestinal microorganisms. The primary polyphenol substrates reduced by these microorganisms include ellagic acid, caffeic acid and isoflavones.Several intestinal microorganisms responsible for these reactions were identified [74]. For example, the sapogenins of soya bean glycosides are reduced to dihydrodaidzein by a strain of Coprobacillus called MRG-1. The dihydrodaidzein is then hydrogenated to estradiol by the Eggerthella strain [75]. Intestinal bacteria can remove hydroxyl groups from phenolic molecules in a particular way. This reducing property has been intensively studied in the catabolism of ellagic acid, which is involved in the production of different urolithins. There are specific metabolic phenotypes responsible for the synthesis of urolithin, and the bacterial species involved in this metabolic transformation have been identified and demonstrated [76].

In summary, the factors that influence the metabolic degradation of dietary polyphenols by the intestinal flora include two main points: the structure of the polyphenols and the type of microorganism. Taking the structure of polyphenols as an example, in vitro experiments revealed that the rate of freeing bound Ferulic acid in fermented wheat-bran dietary fibre was significantly lower than that of bound Ferulic acid in sugar beet fibre under the same conditions, mainly due to structural differences in the bound polyphenols [77]. As an example, it has been shown that the metabolic phenotype of equol in humans is determined by the microbial composition of the gut, with equol producers having microorganisms such as *Streptococcus intermedius*, *Ruminococcus productus* and *Bacteroides ovatus* in their gut, while the guts of non-equol-producers lack these bacteria [78].

### 3.2. Regulation of Intestinal Flora by Dietary Polyphenols

As dietary polyphenols regulate gut microbiota, they are essential in maintaining a healthy and balanced gut microbial composition and diversity. The main functions of dietary polyphenols include enhancing intestinal nutrient uptake, improving immunity, anti-tumour effects, and promoting metabolism. Dietary polyphenols also influence the transformation of the gut flora into metabolites by affecting the enzyme lines produced by the gut flora, thereby regulating the gut flora [79]. Recent studies have shown that dietary polyphenols are associated with cell signalling, gene regulation pathways and the ability to regulate gut microbiota. For example, polyphenols can affect the *Faecalibacterium*/*Bifidobacterium* (F/B) ratio by inhibiting the growth of specific bacterial species. A series of pharmacological effects of different phenolic compounds has been demonstrated [80]. However, the health effects of these compounds depend on their bioavailability (extent of absorption, metabolism and excretion from the body). Quercetin can specifically proliferate *Bifidobacteria* in the intestines, thus making the intestinal environment acidic, inhibiting the proliferation of harmful flora and promoting bowel peristalsis [81]. Anthocyanins can regulate gut flora structure and promote the body’s absorption of minerals and lipid metabolism [82]. Dietary polyphenols can be digested in the gut as SCFAs, which are responsible for immune enhancement, inflammation and metabolism. They can accumulate in the colon, influencing and altering intestinal microbiota composition.

Related polyphenols can be broken down and absorbed by beneficial bacteria in the gut and promote the growth and reproduction of beneficial bacteria. The ingestion of polyphenols may also benefit the host by inhibiting the growth of harmful bacteria through regulating the gut microbiota [83]. Available in vitro and in vivo results show that polyphenols can selectively inhibit pathogenic intestinal bacteria, promote the growth of beneficial bacteria and reduce the F/B ratio, thus optimizing the structure of the intestinal flora and having a positive impact on health [84]. Flavonol causes an increase in Lactobacillus and *Bifidobacteria*. After the consumption of anthocyanins, the growth and reproduction of probiotics (*Lactobacillus*, *Bacillus* and *Heterobacter*) are induced. A study on the effects of the phenolic components of grape pomace on the intestinal microbiota of rats found that constructing a model of long-term administration selectively regulated the intestinal microbiota to a healthier phenotype, resulting in a higher proportion of probiotics and lower levels of *Clostridium perfringens*. The in vitro fermentation of chlorogenic acid, caffeic acid, rutin and quercetin significantly increased the production of propionic and butyric acids, with the highest increase in butyric and propionic acid production demonstrated by caffeic acid [85]. The dietary intake of polyphenols is fermented by symbiotic bacteria in the intestines, resulting in SCFAs including formate, acetate, butyrate, and propionate. SCFAs are beneficial to hosts not only because they have antibacterial properties and maintain the integrity of the intestinal layer but because SCFAs also protect the intestines by reducing morphological defects and preventing lipopolysaccharide-induced autophagy. *Lactobacillus* and the SCFA producer *Alistipes* are abundant in intestinal flora with polyphenol intake, while *Prevotella* is less abundant [86].

The intake of specific food components can also affect changes in the gut microbiota, helping to maintain the metabolism, REDOX and immune homeostasis in the colon, thus maintaining the whole body. In recent years, much experimental evidence has been accumulated that dietary phenols are a class of food molecules that can influence this change [87]. Tea polyphenols are prebiotic to the gut microbiota, as they increase the number of potentially beneficial bacteria (e.g., *Bifidobacteria* and *Faecobacteria*) and reduce the number of potentially harmful bacteria (e.g., *Myxobacteria* and *Bacillus*). Tea polyphenols have long been known to inhibit the growth of a wide range of pathogenic bacteria. It is agreed that tea phenols increase the overall microbial diversity, promoting *Bifidobacterium*, *Lactobacillus*, *Ackermannia* and Bacillus and inhibiting Clostridium [88]. In addition, a study on tryptophan metabolism groups showed that dietary supplementation with tryptophan increased the abundance of *Lactobacillus* in the gut. *Lactobacillus*, a bacterial genus capable of transforming tryptophan, converts tryptophan to indole aldehyde and indole-lactic acid through aminotransferase and indole lactate dehydrogenase. Prebiotics reach the colon, selectively fermented by glycolytic bacteria [89].

### 3.3. Mechanisms of Dietary Polyphenol Intervention in the Central Nervous System

The intake of specific food components can also affect changes in the gut microbiota, helping to maintain the metabolism, REDOX and immune homeostasis in the colon, thus maintaining the whole body. In recent years, much experimental evidence has been accumulated that dietary phenols are a class of food molecules capable of influencing this change [87]. Tea polyphenols are a prebiotic for the gut microbiota as they increase the number of potentially beneficial bacteria (e.g., *Bifidobacteria* and *Faecobacteria*) and reduce the number of potentially harmful bacteria (e.g., *Myxobacteria* and *Bacillus*). Tea, with a diverse range of phenolic compounds, provides a rich treasure trove of resources for preventing and treating neurological disorders. Studies have shown that dietary polyphenols have various functions, such as inhibiting amyloid β-protein aggregation, reducing amyloid β-protein production, reducing neuroinflammation, protecting nerve cells and inhibiting acetylcholinesterase activity [90]. Plant polyphenols are stable and are not absorbed by various intestinal enzymes. Plant polyphenols interact with intestinal microorganisms to produce several beneficial nutritional factors for the body [91]. Polyphenols may reduce inflammation in Th1 and Th17 through direct and indirect effects on T cells. In contrast, the human leukocyte antigen (HLA) class II-mediated immune recognition of malignant B cells can be enhanced. Resveratrol can alter the process of Th17 differentiation. Indeed, resveratrol deacetylates the transcription factor STAT3, and resveratrol and other polyphenols can act in inflammatory diseases of the CNS by blocking interleukin-related Th17 and disrupting Th17 polarization [92]. In addition, resveratrol increases the number of *Murine Bifidobacteria* and *Lactobacilli*, promotes the metabolism of amino acids, nucleotides and unsaturated fatty acids, increases the levels of the cytokines IL-10, IL-8 and C-reactive protein and activates NK cells [93].

At the same time, metabolism produces short-chain fatty acids (such as acetic acid, propionic acid, butyric acid and LBA) that lower the pH of the intestinal system, thereby inhibiting the production of harmful bacteria (such as *Bacteroides*, *Clostridium* and *E. coli*) [94]. On the other hand, propionibacterium regulates the synthesis of vitamin B12 from cbiMCbl ribose, and most butyric and propionic acids are metabolites of Roseburia in the intestinal flora. Butyrate acts on GPR109a receptors in microglia and reduces NLLP-3-mediated IL-1β in the microglia. G-protein-coupled receptors enhance IL-10 secretion in the antigen-specific Th1 cells of mice with colitis, thereby reducing neuroinflammation [95]. Most plant polyphenols enter the body and interact directly with the intestinal flora in both directions, influencing the development of the intestinal and nervous systems and improving the function of the intestinal barrier and BBB [96]. Various natural plant ingredients also act on the intestinal flora to maintain the stability of the nervous system. For example, human milk oligosaccharides (HMOs) and Splenda can reduce *Clostridium perfringens* and increase IL-10 and IL-6. As a result, inflammation is prevented, immunity is improved, oxidative stress is reduced and neurological structures are protected [97]. Ellagitannins, important dietary polyphenols, are found mainly in walnuts, berries and pomegranates. Their metabolites, ellagic acid and urolithins, are involved in many key signalling pathways, antioxidant and anti-inflammatory activities and the function stabilization of the intestinal barrier and BBB through their action on the gut–brain axis [98]. In vitro investigations on BV2 microglia cells showed that urolithins significantly reduced the production of proinflammatory cytokines (TNF-α and IL-6) via the activation of SIRT-1 and autophagy initiation [99], attenuated neuroinflammation, reduced inducible nitric oxide synthase gene expression, and the suppression of JNK/c-Jun pathway activation [100]. In addition, urolithins suppressed NF-κB activity and the phosphorylation of JNK, ERK and Akt and mRNA levels of proinflammatory TNF-α, IL-6, IL-1β, iNOS and COX-2 genes [101] and increased the production of the anti-inflammatory cytokine IL-10 and the phosphorylation of AMP-activated protein kinase (AMPK), which is associated with anti-inflammatory and antioxidant activities [102]. Ellagic acid regulated intestinal microbiota through increasing *Lactobacillus* species and decreasing *Escherichia coli* levels [103], enhanced the immune response by modulating different signalling pathways and increasing the intestinal mucosal barrier function via activating Zonula occludens-1 and Occludin proteins, which are implicated in the tight junction structure of enterocytes and intestinal permeability [104]. Following polyphenol treatment, mitochondrial dysfunction and the levels of some neuroinflammatory biomarkers were reduced in mouse brains, and dendritic spines and lengths were significantly increased [105]. Moreover, in the hippocampus, the Nrf2/HO-1 pathway was activated, and the levels of IL-1β, IL-6 and TNF-α were reduced [106]. Flavonoids modulate SCFA, influence the development of the CNS and BBB, reduce cortisol levels in the VN and significantly impact the treatment and prevention of neurological disorders [107]. Gao et al. found that Cistanche also improved cognitive performance by maintaining the stability of the gut microbiota through D-galactose, thereby resisting oxidation and inflammation [108]. The metabolite of sesamin in the host body is lactone. Sesamin also increases the abundance of *Bacillus mimics* and *S24-7 Bacteria*, reorganizes the intestinal microbiota with lipopolysaccharide inhibition, prevents neurological inflammation and thus aims to protect the barrier integrity of the intestinal system and effectively inhibit glial cell activity [109]. The antidepressant effect of tea polyphenols was confirmed by the tail suspension test and forced swimming test in mice. Tea polyphenols and their metabolites increase intestinal probiotics and inhibit harmful bacteria by interfering with the growth and metabolism of the intestinal microbiota, energy metabolism and the normal function of cell membranes. Tea polyphenols significantly reduced pro-inflammatory cytokines (TNF-α, IL-6 and IL-12) and increased anti-inflammatory cytokine IL-10 in lipopolysaccharide-stimulated RAW 264.7 macrophages and a DSS-induced colitis mouse model, with anti-inflammatory effects that modulated anxiety-like behaviour and improved sleep [110].

Dietary polyphenols modulate the gut microbiota in subjects, helping to maintain gut homeostasis and improving neurodegenerative diseases caused by ageing. In animal AD models, curcumin treatment improved cognitive performance by reducing the burden of amyloid plaques in the hippocampus. Similarly, curcumin intake reduced the levels of *Bacillariophyceae*, *Prevotella* and *Lactobacillariophyceae*, all associated with AD development [111]. Epigallocatechin gallate (EGCG), the most abundant component of catechins, has potent antioxidant and anti-inflammatory activities, reduces oxidative stress damage and is considered an effective compound for preventing and treating AD. Xu et al. found that green tea polyphenols improved spatial cognitive deficits, scavenged oxygen free radicals, enhanced antioxidant capacity, reduced lipid peroxide production and reduced oxidative DNA damage in a rat model of chronic cerebral hypoperfusion [112]. Zaplatic et al. constructed an AD transgenic mouse model and subjected it to quercetin feeding for three months. The results showed that quercetin intake reduced amyloid β-protein deposition in the hippocampus and amygdala, decreased Tau protein phosphorylation, inhibited astrocyte and microglia proliferation and improved cognitive deficits in the mice [113]. The analysis of the plant polyphenol mouse model shows that polyphenols can reverse the cognitive defect of AD and reduce the risk of AD.

**Figure 2:**
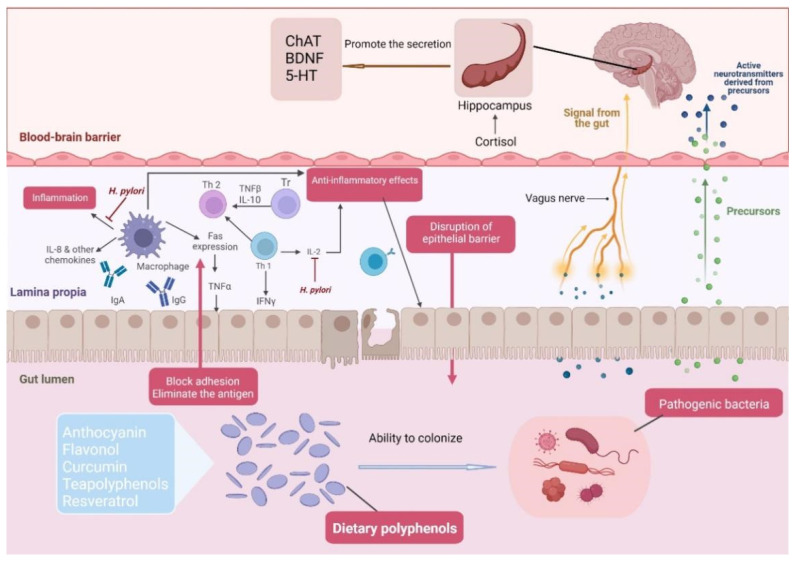
Dietary polyphenols promote the growth and reproduction of probiotics, maintain intestinal micro-ecological balance, improve immunity, induce gut-brain neurotransmitter signalling and stimulate the hippocampus to protect the health of the central nervous system.

Dietary polyphenols are also influential synchronization factors which regulate the expression and oscillation rhythm of biological clock genes and affect the internal environment. Chararya Palinurus flavonoid (CPF) is a flavonoid that has an antioxidant effect on the anti-gallic acid component, which can remove oxygen radicals and free radicals and inhibit lipid peroxidation and ROS production. Phenolic compounds inhibit the expression of inflammatory factors so that more differential expression genes (DEGs) are enriched in KEGG pathways and PI3K-Akt signalling pathways. CPF positively affects circadian rhythm regulation, affects the expression of clock genes, treats metabolic syndrome caused by circadian sequence disorder and improves host microecological health [114]. The regulation of circadian rhythms by plant polyphenols is mainly concentrated on tea polyphenols and peach polyphenols. Tea polyphenols can proliferate against probiotics through cell targets via hydrogen bonding, hydrophobic interactions between covalent bonds and protein formation [115]. Tea polyphenols may bind to cell membranes, thus interfering with cell membrane function and inhibiting bacterial growth. After the host intake of tea polyphenols, the abundance of acetate and butyrate in the body increases and promotes the production of SCFAs, affecting metabolism and cell growth [116]. According to the results of individual cell transcription in the hypothalamus, tea polyphenols increase the abundance of glial cells in the host. IThey improves the mutation of clock genes (Csnk1d, Clock, Per3, Cry2 and BhIhe 41) caused by circadian rhythm disorder [34]. In regulating circadian rhythm, tea polyphenols can reverse gene transcription in the liver and hypothalamus under oxidative stress and affect the expression of translator protein, activating Nrf2/KEAP-1 antioxidant defence signal and regulating the REDOX response in vivo [117]. EGCG has different effects on inhibiting cell differentiation due to different structures. Immediately upon entering the body, the metabolism produces metabolic by-products to improve disturbances caused by circadian disorders [118]. EGCG can improve circadian disturbances caused by abnormal lipid metabolism by controlling the SIRT1-PGC1 ring, thereby significantly increasing the consumption of liver fatty acids and inhibiting liver fatty acid proliferation. The Insig2-steps pathway, a nuclear receptor involved in hepatic cholesterol synthesis and conversion, helps docosahexaenoic acid (DHA) to improve the composition of the intestinal flora, regulate circadian rhythms and influence bile acid metabolism. In contrast, circadian rhythms can reverse the nuclear receptor pathway. Peach polyphenols, similar in structure to tea polyphenols, also have a rhythm-regulating effect. It was found that mice consuming peach polyphenols had increased levels of hypothalamic glial cells and an increased expression of neuroprotective genes (Sox9 and Mbps). Peach polyphenols regulate host biological rhythms by modulating protein activity, which positively affects host biological regulation [19] (Figure 2).

## 4. Conclusions

This review focuses on the bidirectional interactions between the gut microbiota and the brain and their effects on cognition, mood and circadian rhythms. It also discusses the role of dietary interventions in regulating host circadian rhythms and neurocentral systems by the GBA. In recent years, new sequencing technologies have allowed us to better understand the mechanisms of interaction between the gut microbiota and other systems. Various studies have also confirmed the regulatory effect of dietary pattern change on the gut microbiota, which has a series of effects on host cognitive function, emotion regulation and circadian rhythm maintenance through the GBA. However, most cellular or animal studies are carried out on a short-term basis; more long-term and large-scale epidemiological and clinical studies are needed in the future to determine the mechanisms underlying their effects on neurodegenerative diseases. Future methods of improving the bioavailability of dietary polyphenols should be explored. The bidirectional role and metabolic behaviour of their content and intestinal flora should be studied to provide new directions for developing treatments for CNS disorders.

## Data Availability

Data is contained within the article.
